# Diagnosis and Management of Gastrointestinal Neuroendocrine Tumors: A Comprehensive Literature Review

**DOI:** 10.7759/cureus.14006

**Published:** 2021-03-19

**Authors:** Omid Yazdanpanah, Sarvani Surapaneni, Layla Shanah, Sohaip Kabashneh

**Affiliations:** 1 Internal Medicine, Wayne State University Detroit Medical Center, Detroit, USA

**Keywords:** functional neuroendocrine tumor, gastrointestinal neuroendocrine tumor, carcinoid syndrome, gi malignancy, ga-68 dotatate scan, chromgranin, 5-hiaa, peptide receptor radiation therapy(prrt)

## Abstract

Neuroendocrine tumors (NETs) are epithelial neoplasms with predominant neuroendocrine differentiation and the ability to synthesize and secrete variable hormones and monoamines. They are relatively rare, accounting for 2% of all malignancy cases in the United States. The most common system affected by NETs is the gastrointestinal tract. Clinical presentation depends on the organ being involved and the hormone being secreted. It can be variable from asymptomatic incidental findings on imaging to intestinal obstruction, or carcinoid syndrome (CS). Several biochemical testings are developed to help with the diagnosis of NETs including 5-hydroxyindoleacetic acid (5-HIAA) and chromogranin A (CgA). Computerized tomography (CT) scans and magnetic resonance imaging (MRI) are the most commonly used modalities to localize the primary tumor and evaluate for metastasis. However, radionuclide imaging using somatostatin receptor-based imaging techniques has improved accuracy to detect smaller neoplasm. Surgical removal is the mainstay of treatment for locoregional tumors. Several medical managements are available for non-respectable NETs which include SSAs, peptide receptor radionuclide therapy (PRRT), and platinum-based chemotherapy agents.

## Introduction and background

Neuroendocrine tumors (NETs) are a heterogeneous group of malignancies that originate from cells that share similarities with both neurons and endocrine cells. They have dense core granules similar to the granules containing monoamines present in serotonergic neurons and they have the ability to synthesize and secrete monoamines [1,2]. They arise from the endocrine glands located in various parts of the body such as the pituitary, parathyroid, adrenal, thyroid, endocrine cells of the pancreas, gastrointestinal, and the respiratory system [2]. NETs can be classified based on anatomical location or the degree of differentiation [3]. They are also further graded as G1, G2, or G3 based on mitotic count and/or Ki-67 cell labeling index [3,4].

NETs account for 2% of all malignancies with a prevalence of fewer than 200,000 cases in the United States [1,5]. Dasari et al. conducted a retrospective study of 64,971 patients with NET and concluded that the age-adjusted incidence rate increased 6.4-fold between 1973 (1.09/100,000) and 2012 (6.98/100,000) [6]. The incidence of NETs originating from the gastrointestinal tract and pancreas has tripled in the last few decades [4].

The most common organ systems affected by NETs are the gastrointestinal tract and the respiratory tract accounting for 62-67% and 22-27% of the total NETs, respectively [5]. In the digestive tract, the most common sites to be affected by NETs are the small intestine (30.8%), rectum (26.3%), colon (17.6%), pancreas (12.1%), and appendix (5.7%) [7]. Midgut NETs occur predominantly in white patients, while rectal NETs occur more commonly in African American, Asian, and Native American patients [8].

The aim of this article is to review the diagnosis and management of NETs which have had a growing incidence among malignancies in the United States.

## Review

Genetic and environmental factors

The genetic mutations noted in NETs vary based on the anatomic location; pancreatic NETs may have somatic mutations of *MEN1*, *DAXX*, *ATRX*, *PTEN*, and members of the *mTOR* signaling pathway, gastrointestinal NETs may show *CDNK1B* mutations [3,9,10]. Inactivation of *RB1* and *TP53* is often noted in neuroendocrine carcinomas rather than NETs [11]. Familial predisposition is well established in up to 10% of gastrointestinal and pancreatic NETs. A family history of NETs in first-degree relatives increases the risk of their development by 3.6-fold [8]. NETs are associated with syndromes such as multiple endocrine neoplasia 1 (MEN1), Von Hippel-Lindau (VHL), neurofibromatosis type 1 (NF1), and tuberous sclerosis (TS) [12]. No predisposing environmental factors have been established in the literature so far [8].

Clinical presentation

Clinical manifestations of NETs depend on the location of the primary tumor and its functionality. As the majority of NETs are non-functional, they present late usually with symptoms of mass effect or liver metastasis [2,13]. Carcinoid syndrome (CS) develops in patients with metastatic disease, especially those with liver metastasis, and it is present in about 10% of patients with NETs. CS manifestations are flushing (94%), diarrhea (78%), abdominal cramping (50%), valvular heart disease (50%), telangiectasia (25%), wheezing (15%), and edema (19%) [14,15].

Flushing associated with CS is distinctive, reddish-brown with variegated margination that manifests as wheals over the entire body including palms and soles. It is usually triggered by foods and adrenergic stimuli such as pain and anger [16]. Diarrhea in CS is explosive, watery, non-bloody, and accompanied by abdominal cramping [17].

Serotonin stimulates fibroblast growth and fibrogenesis leading to deposits of fibrous tissue on the endometrium, valves, cardiac chambers, and pulmonary and aortic arteries, leading to right valvular heart disease. The left side is usually spared due to the inactivation of humoral substances by the lung. Additionally, mesenteric fibrosis can develop leading to clinically significant intestinal ischemia and/or obstructive uropathy [18-20].

Redirection of dietary tryptophans for the synthesis of large amounts of serotonin can cause niacin deficiency which manifests as pellagra. Symptoms of pellagra include rough scaly skin, angular stomatitis, glossitis, diarrhea, and encephalopathy [21].

The presentation of pancreatic NETs (PNETs) depends on the type of hormone being secreted by the tumor. Insulinomas are typically small, benign, and present with episodic hypoglycemia. Pancreatic gastrinomas are usually malignant, 25% are associated with MEN1. They typically present with peptic ulcer disease and diarrhea. Glucagonomas are associated with a clinical syndrome that includes diabetes, a characteristic rash (necrolytic migratory erythema), cheilitis, anemia, weight loss, diarrhea, venous thrombosis, and neuropsychiatric symptoms. Watery diarrhea, hypokalemia, and hypochlorhydria are the main features of VIPomas [22,23]. Non-functional in 40% of cases, PNETs can present later in the course of the disease with local compressive symptoms or metastatic disease [24].

Diagnostic evaluation

Several biochemical tests have been used for the diagnosis of CS. 5-Hydroxyindoleacetic acid (5-HIAA) is the end product of serotonin metabolism. Measurement of 24-hour urinary excretion of 5-HIAA has a sensitivity and specificity of more than 90%, therefore, it is a practical initial diagnostic test. However, its sensitivity is low in individuals with NETs without CS [14,25]. 5-HIAA can become falsely positive due to certain drugs such as acetaminophen, guaifenesin, nicotine, and mesalamine. It can also be affected by tryptophan/serotonin-rich foods such as bananas. These confounders should be avoided at least three days prior to testing [26].

Chromogranin A (CgA) is a glycoprotein secreted by NETs. Marked elevation of CgA can occur with the use of proton pump inhibitors (PPIs) or in conditions like atrophic gastritis, inflammatory bowel disease (IBD), irritable bowel syndrome (IBS), renal failure, hyperthyroidism, heart failure, hypertension, or prostate cancer. Moreover, the lack of international standardization, makes it hard to infer one CgA assay to another. This test has poor specificity and is not recommended as a screening test. However, it is an appropriate marker for patients with established disease in order to assess disease progression and response to treatment [27-29].

Several radiographic studies are available for localizing NETs. Triple-phase computerized tomography (CT) scans and magnetic resonance imaging (MRI) are the most commonly used modalities to localize the primary tumor and evaluate for metastasis. Somatostatin receptor imaging modalities have the highest sensitivity and specificity for detecting NETs. High levels of somatostatin receptor (SSTR) expression on most well-differentiated NETs allows for the detection of these tumors through whole-body imaging. Octreotide scans can visualize SSTR-expressing tumors using 111-In pentetreotide. The accuracy was improved by adding single-photon emission computed tomography (SPECT) to planar imaging [30,31]. The newest positron emission tomography (PET) tracers for SSTR imaging such as 68-Ga Dotatate, in combination with CT, have remarkably increased the sensitivity [32,33].

Management

Surgical Management

Surgery is the mainstay of treatment for local and locoregional gastrointestinal NETs. Type of the surgery changes based on the location of the NET. Indications for surgery in NETs based on European Neuroendocrine Tumor Society (ENETS) guidelines are described in Table 1 [8,34-36].

**Table 1:** Indications for surgical management of neuroendocrine tumors.

NETs origin	Indications for surgery	Recommended procedure
Pancreatic NETs	Symptomatic, intermediate‐to‐high grade, or size greater than 2 cm	Whipple resection or distal pancreatectomy/splenectomy
	Nonfunctional, size less than 2 cm	Controversial but ENETS recommends watch and wait surveillance approach
Midgut NETs	Jejunal or proximal ileal NETs	Partial small bowel resections
	NETs in or near the ileocecal valve	Right hemicolectomy
Appendiceal NETs	Size less than 1 cm	Simple appendectomy recommended
	Size 1 to 2 cm	Right hemicolectomy can be considered
	Size greater than 2 cm	Right hemicolectomy recommended
Colorectal NETs	Colon NETs	Partial colectomy
	Rectal NETs with size less than 2 cm	Endoscopic resection or trans anal excision
	Rectal NETs with a size greater than 2 cm	Low anterior resection or abdominoperineal resection

Medical Management

In recent years, the new systemic treatments for tumor and syndrome control have succeeded to delay the progression of the disease as well as decreasing symptoms related to hormone secretion [8].

Somatostatin analogs (SSAs) such as lanreotide and native human somatostatin are similar in pharmacodynamics but differ in pharmacokinetics as SSAs have a longer half-life [8]. The ENETS currently recommends medical management with first-line SSA for patients with a small intestinal or pancreatic NET with a Ki67 tumor index of 10% or less based on landmark trials including the PROMID trial and the CLARINET trial. ENETS also currently recommends perioperative octreotide prophylaxis (octreotide 250-500 μg subcutaneously or intravenously) in patients with CS to prevent intraoperative carcinoid crisis [37-40]. Telotristat ethyl is a medication that inhibits tryptophan hydroxylase, a rate-limiting enzyme in the conversion of the amino acid tryptophan to serotonin. It is a generally safe and well-tolerated additional option for patients whose symptoms are not adequately controlled by SSAs [41].

**Figure 1:**
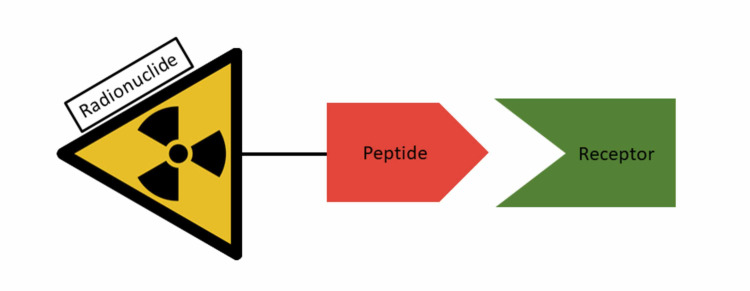
Schematic mechanism of action for peptide receptor radionuclide therapy

Peptide receptor radionuclide therapy (PRRT) or radiolabeled SSAs are a form of targeted radiotherapy against cells that have high levels of somatostatin receptors. PRRT (Figure 1) contains a radionuclide isotope such as lutetium‐177 (177Lu), a peptide (usually octreotide or octreotate), and a linker [42].

The NETTER-1 phase 3 randomized controlled trial compared PRRT with octreotide. Over 229 patients with metastasized, well-differentiated NETs of the small intestine were enrolled into either lutetium-177 (177Lu)-Dotatate 7.4 GBq every eight weeks with intramuscular octreotide LAR 30 mg group or octreotide LAR 60 mg per four weeks group. This study showed that the rate of progression-free survival (PFS) at the end of 20 months improved from 10.8% in the control group to 65.2% in the 177Lu-Dotatate group (95% confidence interval [CI], 50.0 to 76.8). The study also showed a statistically significant higher response rate of 18% in the treatment group compared to 3% in the control group. Significant side effects included cytopenias such as lymphopenia (09%), thrombocytopenia (02%), and neutropenia (01%). Treatment with 177Lu‐Dotatate is available for advanced GEP‐NETs with evidence of SSTR expression on imaging studies based on the NETTER trial [42].

Everolimus is a mechanistic target of rapamycin inhibitor (mTOR inhibitor). It is approved for advanced metastatic pancreatic NETs, progressive metastatic small intestinal NETs, and nonfunctioning lung and gastrointestinal NETs [38,43]. RADIANT‐3 and RADIANT 4, phase 3 randomized controlled trials, showed statistically significant improvement in median PFS in everolimus as compared to placebo [43]. Based on these trials as described, it is approved for advanced metastatic pancreatic NETs, progressive metastatic small intestinal NETs, and nonfunctioning lung and gastrointestinal NETs. However, the significant side effects include stomatitis, diarrhea, fatigue, hyperglycemia, infections, rash, and peripheral edema which limit its use to clinically advanced NETs [8,38,43].

Sunitinib malate inhibits multiple receptor kinases such as VEGFR and PDGFR and thus has antiangiogenic properties which hinder tumor growth. The SUNNIT trial was conducted comparing Sunitinib to placebo. The primary outcome was the PFS in patients with pancreatic NETs. Sunitinib is currently sanctioned for advanced pancreatic NETs [44].

The ENETS guidelines recommend platinum-based chemotherapy for poorly differentiated grade 3 NETs, but it has limited efficacy in NETs with Ki67 of 20-55% [38,45]. Of the platinum-based agents, carboplatin has better efficacy and lower toxicity compared to cisplatin. The combination of cisplatin with etoposide showed higher response rates of 17-67%, but median survival was not improved significantly [45]. For patients who tolerate platinum-based chemotherapy, FOLFIRI (folinic acid, 5‐fluorouracil [5‐FU], and irinotecan) and FOLFIRINOX (FOLFIRI plus oxaliplatin) have recently shown anti-tumor activity [8]. The 2016 ENETS guidelines suggested using streptozocin and 5-fluorouracil chemotherapy in rapidly progressive NETs with a Ki67 of 5-20% [37].

Based on retrospective data, radiofrequency ablation is commonly used for liver metastases smaller than 5 cm and embolization for larger tumors (bland embolization, chemoembolization with cisplatin or doxorubicin, or radioembolization with 90Ytrium). Hepatic transarterial embolization is usually chosen for progressive unresectable liver metastases and can cause postembolization syndrome that presents with abdominal pain, fatigue, and fever [38].

## Conclusions

NETs account for a small proportion of all malignancies, but their incidence is on the rise. The majority presents with mass effect symptoms. In patients with CS, 5-HIAA can be used as an initial test. CgA can be used to assess disease progression and response to treatment. CT and MRI are commonly used to localize the primary tumor and evaluate for metastasis. However, somatostatin receptor imaging modalities have the highest sensitivity and specificity for detecting NETs. Surgery is the mainstay of treatment for localized NETs. Currently, there are several medical options that can be used to treat NETs including SSAs, PRRT, Everolimus, Sunitinib, platinum-based agents.
